# Effectiveness of Motivational Interviewing and cross platform messaging application in improving oral health knowledge, attitude and behaviours among pregnant women- A Randomized Controlled Trial

**DOI:** 10.12688/f1000research.153000.1

**Published:** 2024-08-02

**Authors:** Amitha Basheer N, Praveen Jodalli, Inderjit Murugendrappa Gowdar, Sultan Abdulrahman Almalki

**Affiliations:** 1 Department of Public Health Dentistry, Manipal College of Dental Sciences, Mangalore, Manipal Academy of Higher Education, Karnataka, Manipal, 576104, India; 2 Former Postgraduate student, Department of Public Health Dentistry, Yenepoya Dental College, Yenepoya Deemed to be University, Mangalore, Karnataka, 575108, UK; 3 Faculty, Department of Preventative Dental Sciences, Prince Sattam bin Abdulaziz University, Al Kharj, 11942, Saudi Arabia

**Keywords:** Pregnancy, Oral health, Wellbeing, Motivational Interviewing, Health education, Randomized controlled trial, Maternal and child health

## Abstract

**Background:**

Body goes through significant hormonal and physiological changes during pregnancy, which could be linked to changes in oral health. Many women are unaware of the negative consequences of poor dental health during and after pregnancy, both for themselves and their children. Therefore, this study aimed to assess the effectiveness of Motivational Interviewing (MI) and cross platform messaging application (WhatsApp messenger) for oral health education on the oral health knowledge, attitude and behaviours among pregnant women attending ante natal care.

**Methods:**

A randomized controlled trial was conducted among 84 pregnant women. Simple random sampling was employed to select participants after oral examination. Participants were randomly allocated to two groups (Group 1: Cross-platform messaging application [WhatsApp]; Group 2: MI) using a lottery method. Pregnant women aged 18 years and older, gestational age between 8 and 30 weeks were included. A face-to-face interview and oral examination were conducted to assess baseline knowledge and oral hygiene status. A follow-up examination was conducted after one month of intervention. Inferential statistics, including the chi-square test and independent t-test, were used to compare variables between the two groups.

**Results:**

The mean knowledge score at baseline was comparable between Group 1 (WhatsApp) and Group 2 (MI). However, post-intervention, Group 2 showed a significantly higher mean knowledge score compared to Group 1. Post-intervention, Group 2 exhibited a significantly better oral hygiene status compared to Group 1. Significant improvements in oral health behaviours were observed in Group 2 compared to Group 1 (p < 0.001).

**Conclusions:**

The findings suggested that while both interventions were effective, MI showed superior results in improving knowledge, oral hygiene status, and oral health behaviours. The personalized and client-centred approach of MI enables participants to explore and resolve ambivalence, promoting a deeper understanding of the importance of oral health during pregnancy.

**Registration:** CTRI ( CTRI/2021/09/036407, 10/09/2021).

**List of abbreviations**
CTRI: Clinical Trial Registry of India
IMA: Instant Messenger Application
MI: Motivational Interviewing
MITC: Motivational Interviewing Training and Consulting
OHI-S: Oral Hygiene Index Simplified

## Contributions of the literature

• Current study provided a comprehensive overview on Motivational Interviewing (MI) and its application in healthcare contexts, particularly in promoting behaviour change among pregnant women.
• Study identified the gaps in the current understanding of oral health knowledge, attitudes, and behaviours among pregnant women, as well as the impact of MI and cross-platform messaging applications in promoting health behaviours.

## Background

Pregnancy induces significant alterations in women’s physical, hormonal, and emotional well-being, profoundly impacting their overall quality of life. ^1^ The body undergoes substantial hormonal and physiological transformations during pregnancy, potentially influencing oral health. ^2^ Pregnancy-associated periodontal diseases emerge as significant changes, manifesting through signs like bleeding, redness, and swelling in the gums. ^3^ When dental calculus and plaque are present, changes in endogenous hormones trigger immune and circulatory responses that amplify the inflammatory response. ^4^ Additionally, the physical and emotional demands placed on pregnant women may contribute to lapses in oral hygiene routines, leading to various complications. ^5^ Prioritizing oral hygiene can play a crucial role in preventing or mitigating the impact of hormone-mediated inflammatory changes in the oral cavity. ^2^

Gum bleeding or nausea during the first trimester of pregnancy might cause some women to stop practicing oral hygiene, including brushing their teeth. ^5^ Moreover, over half of pregnant women refrain from visiting the dentist during their pregnancy. ^6^ Suboptimal dental health during pregnancy poses risks for both maternal and foetal well-being, underscoring its significance in prenatal care. ^7^ Improving the dental health of expecting mothers is a proactive measure that should be taken to prevent early childhood cavities.

Providing health education can exert a substantial impact on the health behaviours of individuals and communities, influencing both their living and working conditions. ^8^ The selection and application of proper teaching strategies is critical to the success of health education programmes. Traditional health promotion tactics often focus on giving knowledge and providing recommendations. ^8^ Despite the fact that evidence has shown that conventional health education techniques help certain populations achieve significant improvements in knowledge, attitude, and behaviour, new strategies such as motivational interviewing (MI) have showed promise in helping people adopt better habits. MI has been found to be helpful in areas such as dental health, substance misuse, diet, and exercise as a client-centred technique of enhancing motivation for change by finding and removing barriers to change. ^6^

Many areas of human behaviour and communication have changed dramatically as a result of advances in information technology. ^9^ These shifts have had a significant impact on educational methods. Cross-platform messaging apps keep you connected even if you’re at your desk or on the road, and cloud-synchronized chat logs, contact lists, and settings are essential features. ^10^ With more than two billion active users across 180 nations, WhatsApp is a free standalone instant messaging application (IMA). This was launched in 2009. WhatsApp uses a storable message, allowing health-related information to be given to specific persons, guiding medicine and behaviour, and boosting self-health management. ^11^

Women lack awareness regarding the adverse outcomes associated with inadequate dental health during and after pregnancy, impacting both their well-being and that of their children. Even though getting orthodontic treatment during pregnancy is safe, many pregnant women choose not to receive it, leading to enduring oral health issues. A proactive strategy could involve educating expecting moms about their oral health and the possible effects of untreated dental caries and gingivitis on the developing baby in order to provide preventive remedies. ^12^ There is a notable gap in existing research, as there is a scarcity of studies examining the effectiveness of educational interventions that incorporate Motivational Interviewing and cross-platform messaging applications such as WhatsApp messenger to improve oral health knowledge and behaviours among pregnant women. Consequently, this study aims to fill this void by assessing the impact of Motivational Interviewing and the use of WhatsApp messenger as tools for oral health education. The primary purpose was to assess the impact of these interventions on the oral health behaviours, attitudes, and knowledge of pregnant women attending antenatal care.

## Methods

**Trial registration:** Trial was prospectively registered in Clinical Trial Registry of India with reference number CTRI/2021/09/036407 on 10/09/2021.

### Study setting

A single centred parallel randomized controlled study was carried out at a tertiary care hospital’s Department of Obstetrics and Gynaecology at Yenepoya Medical College Hospital, Yenepoya Deemed to be University, an institute of higher education located in Mangalore, Karnataka, India. 84 pregnant women aged 18 and above, who attended the hospital for prenatal treatment and had gestational ages ranging from 8 to 30 weeks, participated in this randomised controlled trial. Data for the study were collected between February 1st and April 30th, 2022. Before initiating the study, official permission was obtained from the Head of the Department of Department of Obstetrics and Gynaecology at Yenepoya Medical College Hospital, Yenepoya Deemed to be University.

### Sample size

With an effect size of 0.575 derived from a pooled standard deviation of 5.2 as reported in a significant article by Bahri et al., ^12^ the sample size of the research was determined using the G*Power software with a 5% level of significance and 80% power. The total sample size for each group was 38. The final total sample size was modified to 84, with 42 participants in each group, to account for a 10% non-response error rate.

### Sampling procedure

**Figure 1.**
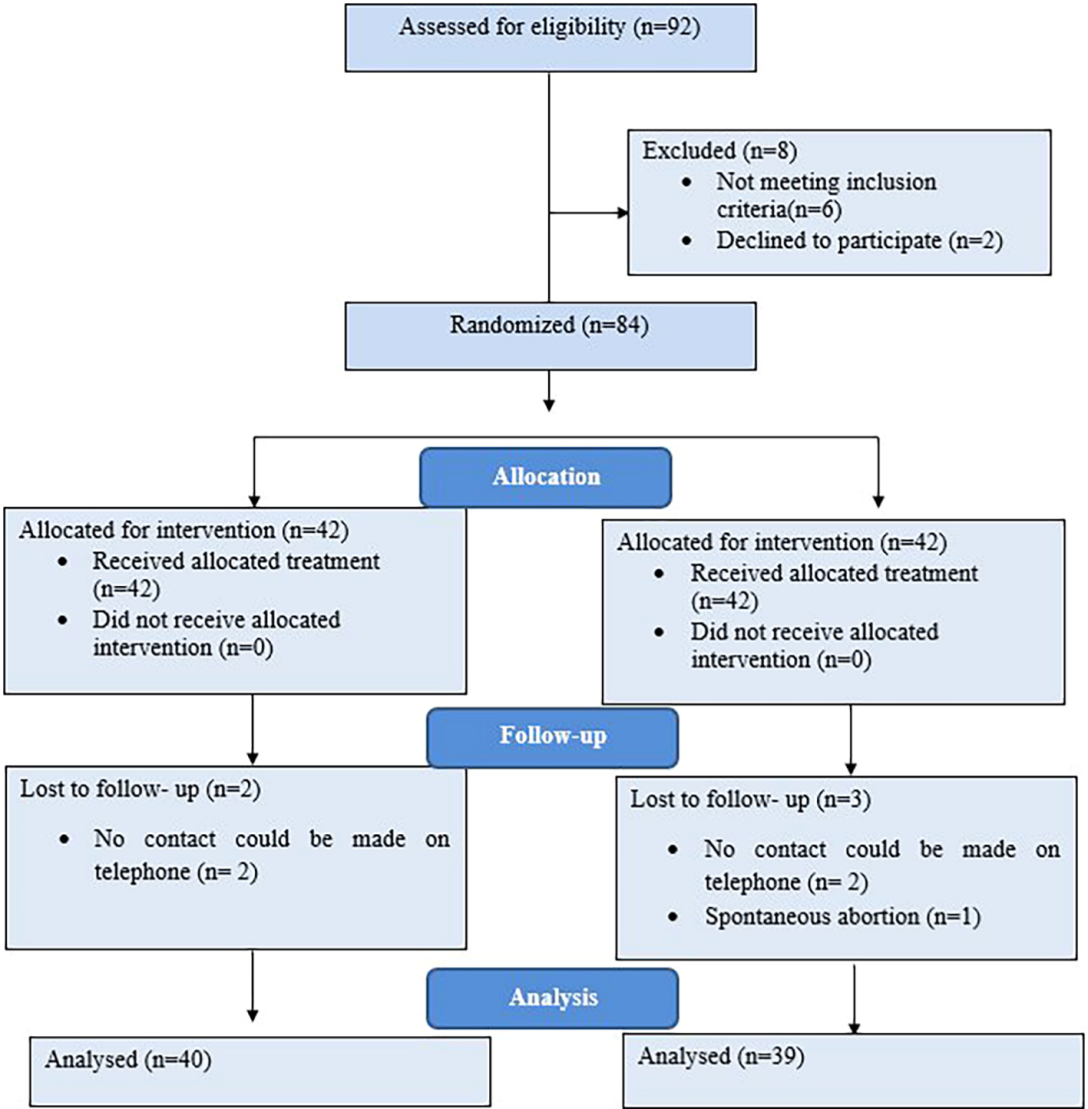
CONSORT flow diagram of the study.

Participants were selected using simple random sampling. Following oral examinations, eligible participants were randomly allocated to two groups using the lottery method. The total sample comprised two groups: Group 1 utilized a cross-platform messaging application (WhatsApp messenger), while Group 2 underwent Motivational Interviewing for oral health education. The enrolment of participants is depicted through the CONSORT diagram ( Figure 1).

### Allocation concealment and enrolment

The random allocation sequence was implemented using sequentially numbered containers. Steps were taken to conceal the sequence until interventions were assigned by ensuring that the containers were opaque and sealed. This process helped maintain the integrity of the randomization process and prevented any potential bias in the assignment of interventions until the participants were allocated to their respective groups. Statistician with expertise in research methodology generated the random allocation sequence but not directly involved in participant enrolment or intervention assignment to maintain the randomness and reduce bias. Research assistant performed participant enrolment. Research assistant explained the study, obtained informed consent, and ensured that participants meet the inclusion criteria. The assignment of participants to intervention groups was carried out by research coordinator (research guide) who was blinded to participant characteristics to ensure unbiased allocation.

### Ethics approval and consent to participate

This study was done in accordance with the principles outlined in the Declaration of Helsinki. Before initiating the study, official permission was obtained from the Head of the Department of Department of Obstetrics and Gynaecology at Yenepoya Medical College Hospital, Yenepoya Deemed to be University. The protocol was reviewed and approved by institutional ethics committee (Yenepoya Ethics Committee-2 of Yenepoya (Deemed to be University), Mangalore, India on 26/02/2021 with reference number YEC2/636. Written informed consent was obtained from eligible participants prior to the study. ^13^

### Inclusion and exclusion criteria

The study included participants who met the following criteria: confirmed pregnancies, aged 18 years and older, with a gestational age between 8 and 30 weeks, and those who provided informed consent. ^13^ Additionally, participants needed to possess a smartphone at the time of allotment and be familiar with the usage of WhatsApp. The minimum educational qualification required was completion of schooling up to the 7th standard, with the ability to read and write in English, Kannada, or Malayalam.

Exclusion criteria comprised individuals with a history of smoking and alcohol consumption. Women with conditions like COVID-19 that could make dental examinations difficult were also excluded from being permitted to participate in the study.

### Data collection

**Examiner training and calibration**

The principal investigator underwent training for Motivational Interviewing from MITC (Motivational Interviewing Training and Consulting), accredited with the American Psychological Association and the New Mexico Counselling and Therapy Practice Board. The format for the interview questionnaire was derived from a study carried out by Bansal et al, (2019). ^14^ Questions were modified and validated by two subject experts. The survey questions were designed to evaluate knowledge, attitude, and behaviours and oral health-seeking behaviour among pregnant women, and the accurate responses to these questions and were provided. ^13^

**Baseline examination**

Data collection involved face-to-face interviews and subsequent oral examinations. The one-on-one interviews were conducted in a designated room where participants comfortably sat on chairs. The primary investigator performed interviews to assess pregnant women’s knowledge, attitudes, and practices related to oral health care. Oral examinations were conducted under natural light, supplemented by torch lights when necessary, and performed by a single examiner with the assistance of an assistant. The OHI-S index ^15^ has been used to evaluate the status of oral hygiene, while The Loe and Silness Gingival Index ^16^ was employed to measure gingival inflammation. Contact information, including WhatsApp numbers, was collected from all participants.

### Intervention

Using both English and their respective regional languages (Kannada/Malayalam), participants in Group I received WhatsApp messages with information on oral health care for expectant mothers and children.

In Group II, participants received a 20-minute talk based on the Motivational Interviewing (MI) approach, delivered by a dentist trained in MI, who also served as the Principal Investigator (PI). The MI approach encompassed four key aspects: Partnership, Acceptance, Compassion, and Evocation. The motivational interviewing process unfolded in two phases. During the first phase, efforts were made to establish rapport, build trust, and identify the problem of concern. The MI provider established rapport by posing open-ended questions concerning participants’ dental health, their child’s oral health, and their aspirations for both. These were questions that addressed several topics, including establishing commitment to change, seeing potential challenges, strengthening commitment to change, and finding challenges to change.

In the second phase, the approach shifted from contemplation to the action stage. Participants were asked to consider the benefits and drawbacks of change, along with potential barriers to action for each proposed change. The focus primarily revolved around collaboratively developing an action plan. The principal investigator effectively communicated with patients using the OARS method, which involves Open-ended questions, Affirmations, Reflective listening, and Summarizing.

Open-ended questions were employed to initiate a conversation between the PI and the patient, encouraging the patient to share thoughts and concerns. Affirmations acknowledged the challenges of behaviour change while highlighting the patient’s strengths to boost self-efficacy. Reflective listening involved actively understanding and comprehending the patient’s statements, fostering a collaborative exploration of behavioural change and motivation. Summarizing demonstrated attentive listening and provided a concise recapitulation of the conversation, aiding in addressing ambivalence.

### Follow up

During the one-month follow-up visit after the intervention, the PI conducted interviews with the participants to re-evaluate their knowledge, attitude, and behavior regarding oral health care, employing the same set of questions used during the baseline examination. The OHI-S index and Gingival Index were once again employed for oral health assessments.

During the study period, 5 participants were lost to follow-up, with 2 participants from Group 1 and 3 participants from Group 2 failing to complete the follow-up process. Consequently, the final sample size considered for analysis comprised 79 participants, with 40 participants in Group 1 and 39 participants in Group 2.

### Outcomes

By evaluating the effectiveness of the two different therapies, the primary outcome of the study was to improve participants’ knowledge, attitudes, and behaviours related to oral health. A questionnaire was employed for this purpose. Enhancing the participants’ oral health status was the study’s secondary outcome, which was evaluated using the Gingival and OHI-S indices.

### Statistical methods used for data analysis

Data recorded in MS-Excel underwent statistical analysis using SPSS version 24.0. ^13^ While frequency and percentage were used to represent categorical variables, mean and standard deviation were used to express continuous variables. Descriptive statistics, including categorical data, were presented as frequency with percentage in brackets and a 95% confidence interval (CI). Fischer exact test/Chi-square test assessed associations between study variables.

For comparisons across groups, the Mann-Whitney U Test was employed, and for comparisons within groups, the Wilcoxon signed-rank test was utilised to assess the pre- and post-intervention measures. The Paired t test evaluated the mean change in knowledge scores within each group before and after the intervention, and the Independent Sample T test was used to compare mean knowledge scores between groups before and after the intervention. P<0.05 was set as a statistically significant.

## Results

The mean age of participants in Group 1 was 25.28 years, while in Group 2, it was 24.26 years, as shown in Table 1. The majority of study participants in both groups had a Pre-University Course (PUC) qualification, with percentages of 32.2% in Group 1 and 41% in Group 2. Additionally, a predominant proportion of participants identified with the Muslim religion, comprising 49.4% of the overall study population. Regarding the gestational period, a majority of participants (62.0%) were in the second trimester ( Table 2). Responses for question 6 and 7 between the groups were included to analyse the attitude of participants towards the oral health. There is no significance (p>0.05) found between the groups for both questions.

**Table 1.** Distribution of study participants based on their age (N=79).

Sl. No	Group	N	Mean Age	SD
1	**1-** Cross platform messaging application (WhatsApp messenger)	40	25.28	±4.16
2	**2**- Motivational Interviewing	39	24.26	±4.48

The data represented is mean and standard deviation.

**Table 2.** Distribution of study participants based on their socio demographic characteristics (n=79).

Socio demographic characteristics		Group		Total	Chi Square Test	
		1	2		Chi Square value	p-value
**Education**	**10** ^**th**^	12(30.0)	08(20.5)	20(25.3)	-	0.63(NS) ^#^
	**7** ^**th**^	01(2.5)	02(5.1)	03(3.8)		
	**Graduate**	08(20)	10(25.6)	18(22.8)		
	**Post Graduate**	06(15)	03(7.7)	09(11.4)		
	**Pre-University College**	13(32.5)	16(41.0)	29(36.7)		
**Religion**	**Christian**	06(15.0)	07(17.9)	13(16.5)	0.42	0.81(NS)
	**Hindu**	15(37.5)	12(30.8)	27(34.2)		
	**Muslim**	19(47.5)	20(51.3)	39(49.4)		
**Trimester**	**1**	08(20.0)	08(20.5)	18(20.3)	0.29	0.86(NS)
	**2**	24(60.0)	25(64.1)	49(62.0)		
	**3**	08(20.0)	06(15.4)	14(17.7)		

The data represented is frequency with (%) percentage in parentheses. Statistical test used: Chi-square test and (#) Fishers Exact test. Level of significance.

* p<0.05 was considered statistically significant. Abbreviations used: NS-non significant p>0.05.

Table 3 presents the comparison of Index scores between the two groups. Before the intervention, the scores for the Debris Index-S and Gingival Index were compared between Group 1 and Group 2, revealing statistically significant differences with p-values of 0.03 and 0.04, respectively. Group 1’s mean debris score was 2.38, while Group 2’s mean score was 2.03. These differences in mean debris scores were statistically significant. Group 2 had a mean score of 2.05. Group 1 had a mean score of 2.33, and the mean gingival score varied significantly between the groups. However, post-intervention scores for the Debris Index-S, Calculus Index-S, OHI-S, and Gingival Index did not show statistical significance (p>0.05).

**Table 3.** Comparison of Index scores between the groups (n=79).

Index		Group	n	Mean (SD)	Range	Median (Q1-Q3)	Mann Whitney U Test	
							U Statistic	p-value
**Debris Index-S**	Before	Group 1	40	2.38 (0.67)	1-3	2(2-3)	573	0.03 *
		Group 2	39	2.03 (0.71)	1-3	2(2-3)		
	After	Group 1	40	1.80 (0.69)	1-3	2(1-2)	695	0.36(NS)
		Group 2	39	1.67 (0.70)	1-3	2(1-2)		
**Calculus Index-S**	Before	Group 1	40	1.67 (0.62)	1-3	2(1-2)	675.5	0.25(NS)
		Group 2	39	1.51 (0.56)	1-3	1(1-2)		
	After	Group 1	40	1.67 (0.62)	1-3	2(1-2)	675.5	0.25(NS)
		Group 2	39	1.51 (0.56)	1-3	1(1-2)		
**OHI-S**	Before	Group 1	40	2.30 (0.65)	1-3	2(2-3)	636	0.11(NS)
		Group 2	39	2.08 (0.62)	1-3	2(2-2)		
	After	Group 1	40	1.67 (0.62)	1-3	2(1-2)	706	0.42(NS)
		Group 2	39	1.56 (0.60)	1-3	2(1-2)		
**Gingival Index**	Before	Group 1	40	2.33 (0.66)	1-3	2(2-3)	601.5	0.04 *
		Group 2	39	2.05 (0.61)	1-3	2(2-2)		
	After	Group 1	40	1.80 (0.65)	1-3	2(1-2)	630.5	0.10(NS)
		Group 2	39	1.56 (0.60)	1-3	2(1-2)		

Statistical analysis was carried out using the Mann Whitney U Test.

* p<0.05 was considered statistically significant. P>0.05 Non Significant, NS.

The comparison of the two groups’ (n=79) knowledge scores is shown in Table 4. Prior to the intervention, there was no statistically significant difference (p>0.05) in the knowledge scores of the participants in the two groups. However, when comparing knowledge scores between the two groups after the intervention, a statistically significant difference emerged with a p-value of 0.006. The mean value for the score after the intervention was 11.08 for Group 1 and 14.08 for Group 2. Furthermore, the change in scores before and after the intervention between the two groups demonstrated statistical significance with a p value of 0.04.

**Table 4.** Comparison of knowledge scores between the groups (n=79).

Knowledge score	Group	N	Mean	SD	Mean Difference	95% Confidence Interval of the Difference		t	df	p-value
						Lower	Upper			
**Before**	**1**	40	5.60	4.11	-0.09	-1.82	1.63	-0.11	77	0.92(NS)
	**2**	39	5.69	3.56						
**After**	**1**	40	11.08	4.37	-3.00	-5.11	-0.90	-2.84	77	0.006 *
	**2**	39	14.08	5.00						
**Change**	**1**	40	5.48	5.83	-2.91	-5.63	-0.19	-2.13	77	0.04 *
	**2**	39	8.38	6.29						

Statistical analysis was carried out using Independent Sample T test.

* p<0.05 was considered as statistically significant. p>0.05. Non-Significant, NS. Abbreviations used: df- degree of freedom, SD-standard deviation.

## Discussion

Pregnancy constitutes a unique physiological state marked by transient adaptive alterations in body structure driven by elevated production of reproductive hormones such as oestrogen, progesterone, gonadotropins, and relaxin. These hormonal effects extend to the oral cavity, resulting in both temporary and irreversible changes, along with pathological modifications. ^17^ Improving dental health in expectant mothers is not only beneficial to their general health but also essential to the oral health of their unborn child. Pregnancy serves as a “teachable time” for women motivated to alter behaviours linked to adverse pregnancy outcomes. The prenatal care team plays a crucial role in encouraging women to adopt good dental hygiene practices, seek oral health expertise, and complete necessary treatments during pregnancy. Integrating oral health treatment into prenatal care is essential for all pregnant women. ^18^

Motivational Interviewing emerges as a significant therapeutic approach with diverse applications in healthcare settings, emphasizing collaborative therapy engagement and respecting the patient’s autonomy. ^19^ Motivational Interviewing aims to empower patients by fostering their self-awareness regarding the need for change and encouraging a voluntary expression of willingness to change. The approach emphasizes autonomy, allowing individuals to arrive at their decisions to change without being directed or coerced by a health practitioner. ^20^

In recent years, there has been growing attention towards the widespread utilization of modern technology, including cell phones and the internet. Cell phone-based health interventions, particularly those utilizing cross-platform communications, have proven effective for medical and public health purposes. WhatsApp stands out among health education intervention apps due to its ability to provide users with more social information compared to traditional SMS text messaging ^21^^–^^23^

### Oral health and socio-demographic correlates

This study examined into the effects of sociodemographic characteristics on pregnant women’s knowledge, attitudes, and behaviours related to oral health. Age, level of education, and religion, however, did not show statistically significant differences in oral health knowledge, attitude, behaviour, or oral hygiene. Conversely, El-Mahdi Ibrahim et al. ^24^ found a strong correlation between age and dental health as well as attitude towards oral health. They also found that there was a strong correlation between education and oral health habits and oral health knowledge. Similarly, Avula et al. ^25^ found statistically significant results regarding the educational qualifications of subjects. Women in different trimesters did not show significant variations in knowledge, which is in line with research by Pentapati et al. ^26^ Furthermore, research by Mart nez-Beneyto et al. ^27^ and Bamanikar et al. ^28^ found a correlation between pregnant women’s educational attainment and dental health.

### Oral health knowledge, behaviour, and attitude

Understanding pregnant women’s oral health knowledge is essential considering the growing global burden of oral diseases. Their depth of knowledge has a direct impact on the oral health care they provide their children as the primary carers. Furthermore, mothers’ active involvement is crucial in influencing their children’s oral health behaviours, which has long-term benefits. In the current study, pregnant women, who make up a significant fraction of the sample, were found to have overall inadequate oral health awareness, indicating that there is a need to improve their understanding. According to a recent study, pregnant women have very little awareness about dental health. Among the participants only 15.2% were aware about the reason for gum disease was due to poor oral hygiene. It might be because, during their routine check-ups, most of them did not receive advice about the significance of dental health during pregnancy from an oral health team or a gynaecologist. The majority of the study subjects (53.2%) exhibited unawareness of other oral hygiene aids, which contrasts with findings from a study by George et al., ^29^ where it was reported that 83% of respondents used other oral hygiene aids. This study indicates a knowledge gap among participants on the association between impaired oral health and unfavourable pregnancy outcomes, as well as the interconnected connection between pregnancy and oral health. This result is consistent with studies by Avula et al., ^25^ and Gupta et al. ^30^ Therefore, intensified education efforts are essential to enhance knowledge and alleviate suffering in this regard.

In a study conducted on the Indian population, parents’ attitudes toward child dental health were reported to be unfavourable, accompanied by poor dental awareness and knowledge. ^30^ Notably, participants who underwent Motivational Interviewing demonstrated enhanced knowledge across a broader spectrum of knowledge items compared to those who received oral health education through WhatsApp messages. This can be due to WhatsApp is flooded with random messages from relatives/friends and people have a common tendency to ignore lengthy messages. Mothers are usually conceived of as the caretakers for their children’s oral health, and the oral health outcomes of early childhood are influenced by their knowledge, beliefs, attitudes, and behaviour related to oral health.

Oral health education strategies targeting the enhancement of parental oral health knowledge have demonstrated effectiveness in improving pregnant women’s knowledge, attitudes, and behaviours. However, Motivational Interviewing has been shown to be particularly successful in this regard.

In addition, the results of this study showed certain general trends that are noteworthy even though they are not statistically significant. The first was a general lack of knowledge, attitude, and practice about oral health issues for children, including the ideal times to introduce a child to the dentist, start brushing their teeth, and bottle feed them at night.

### Oral health seeking behaviour

The data showed that just 17.7% of the participating women had seen a dentist during the previous six to twelve months, with dental pain or issues pertaining to the teeth, gums, or mouth being their primary reason of consultation. None of the participants sought dental care for routine check-ups, and the majority (30.4%) never went to the dentist. This pattern is consistent with research conducted in the United States, which found that even in circumstances in which oral health issues were present, fewer than fifty per cent of pregnant women visited a dentist. ^31^ The literature has identified several reasons why pregnant women may choose not to seek dental care, which includes lack of time, unsatisfactory dental care, negative attitudes towards dental care providers, negative relationships with spouses, and perceptions of dental experiences. ^32^

### Oral hygiene status of expectant mothers

31.6% of participants in the current study exhibited poor oral hygiene, compared to the 55.7% who showed fair oral hygiene. In a study by Gupta et al., ^33^ comparable outcomes have been observed. All participants had their Oral Hygiene Index-Simplified evaluated, and the results showed that 15.3% had good oral hygiene, 40.7% had fair oral hygiene, and 44.0% had poor oral hygiene.

A different study conducted in Belgaum, India, by Kashetty et al. ^34^ discovered that the pregnant group had a significantly higher mean OHI-S status (2.68). The current study found that prior to the intervention, a greater percentage of pregnant women (55.7%) had poor oral hygiene. This indicates a considerable level of oral hygiene neglect. Pregnancy-related gingivitis may also intensify discomfort while brushing and regular dental care, which could accelerate the build-up of debris and calculus. ^35^ Oral hygiene status of the participants improved after the intervention. 46.8% of the participants showed fair debris score after the intervention. Calculus score didn’t show much improvement after the intervention since the time of the study was only one month. This time is not enough for change in calculus score. 55.7% of the participants had fair oral hygiene score after the intervention.

### Gingival assessment of expectant mothers

Before the intervention, a higher proportion of pregnant women experienced a moderate form of gingivitis, and 31.6% of the participants had severe gingivitis. After the intervention, the percentage of participants with severe gingivitis decreased, with only 12.5% in Group 1 and 8.9% in Group 2 exhibiting severe gingivitis.

In a study conducted by Kashetty et al. ^34^ in Belgaum city, Karnataka, a higher proportion of pregnant women (66.6%) suffered from severe gingivitis. The presence of pregnancy gingivitis might contribute to increased difficulty in brushing and regular dental care, potentially accelerating the deposition of local irritants like debris and calculus. ^36^ The intervention in your study appears to have contributed to a reduction in the severity of gingivitis among the participants.

Considering this was the first study of its kind in the Indian population to evaluate women’s level of understanding regarding oral health as well as their attitudes and behaviours concerning dental health, the study’s strength is its novelty.

The study also investigates the impact of two distinct oral health education methods (Motivational Interviewing and Cross-platform messaging using WhatsApp) on women’s knowledge acquisition and improvement in oral health and hygiene.

However, the study has limitations. It relies on self-reported data, introducing potential biases inherent in this method, such as misclassification of the questions being asked. Additionally, most women completed the face-to-face questionnaire within a short time frame (5 to 10 minutes), and questions pertained to the last year, posing a risk of recall bias. Chance of social desirability bias was present since the method of data collection was face to face interview, it might cause might result in under reporting also. Study duration was too less to assess the change in Calculus index score and OHI-S score. There is a common tendency to ignore lengthy WhatsApp messages, so it might have affected the knowledge score after intervention. The study acknowledges certain limitations, particularly in terms of the sample size. It suggests that a longitudinal study involving a larger number of participants would be desirable to provide more robust and comprehensive findings.

## Conclusion

Maintaining good dental health throughout pregnancy is beneficial for the expecting mother’s health as well as the well-being of the unborn child. Despite these possible advantages, the current study shows that pregnant women lack knowledge and awareness about oral health, with a significant percentage unaware of the consequences of continuing poor dental hygiene during pregnancy. It is necessary to recognise pregnancy as a “teachable” phase, offering a chance to encourage women to take up healthy practices. To promote improved oral health outcomes for the mother and the new-born, it is essential that women as well as their families receive guidance emphasising the importance of dental care during pregnancy.

Both educational interventions in this study resulted in improved scores, with participants who received Motivational Interviewing showing a significant increase in oral health knowledge, along with positive changes in attitude, behaviour, oral hygiene, and oral health status post-intervention. Thus, it is critical to employ a variety of health promotion strategies throughout pregnancy to motivate and inform pregnant mothers about the significance of maintaining optimal dental health. Furthermore, integating discussions about oral health into obstetric care can potentially enhance patients’ awareness and prompt them to schedule dental visits. The demand for innovative, consistent, and comprehensive public health communication strategies aimed at reaching women and promoting oral health in a timely and accessible manner is evident.

## Ethics and consent

This study was done in accordance with the principles outlined in the Declaration of Helsinki. Before initiating the study, official permission was obtained from the relevant authorities.

The protocol was reviewed and approved by institutional ethics committee (Yenepoya Ethics Committee-2 of Yenepoya (Deemed to be University), Mangalore, India on 26/02/2021 with reference number YEC2/636. Written informed consent was obtained from eligible participants prior to the study. ^13^

**Consent of publication:** Not applicable

## Authors’ contributions

ABN: Conception of the work, data acquisition, interpretation of data, drafted the work

PJ: Conception of the work, data analysis, revised the draft, supervision

## Data Availability

Figshare: Effectiveness of Motivational Interviewing and cross platform messaging application in improving oral health knowledge, attitude and behaviours among pregnant women- a Randomized Controlled Trial. https://doi.org/10.6084/m9.figshare.25116926. ^13^ This project contains the following data:
- EXCEL SHEET.xlsx (underlying data) - Additional file 2 CONSORT-2010-Checklist.doc (CONSORT checklist) - INFORMED CONSENT FORM.docx - QUESTIONNAIRE AND CORRECT ANSWERS.docx Data are available under the terms of the Creative Commons Attribution 4.0 International license (CC-BY 4.0).
