# Supplementary material for: Effectiveness of Motivational Interviewing and cross platform messaging application in improving oral health knowledge, attitude and behaviours among pregnant women- A Randomized Controlled Trial
Source: F1000Res. 2024 Aug 2;13:871. [Version 1] doi: 10.12688/f1000research.153000.1 (PMC11387929; doi:10.12688/f1000research.153000.1)
Supplement: Peer review history

# Reviewer report: Reviewer response for version 1

**DOI:** 10.5256/f1000research.167832.r319432

**Published:** 2024-09-26

**Authors:** Ankita jain

**Affiliations:** 1 Teerthanker Mahaveer Dental College and Research Centre, Moradabad, Uttar Pradesh, India

The manuscript titled "Effectiveness of Motivational Interviewing and Cross-Platform Messaging Application in Improving Oral Health Knowledge, Attitude, and Behaviors Among Pregnant Women: A Randomized Controlled Trial" presents a timely and relevant investigation. The study's design is robust, employing a randomized controlled trial approach, which strengthens the validity of the findings. Both motivational interviewing and the use of a messaging application show promising potential for enhancing oral health awareness in this vulnerable population. The authors effectively address the research questions and provide clear implications for practice. I recommend acceptance, with minor revisions for clarity in methodology and data presentation.

Is the work clearly and accurately presented and does it cite the current literature?

Yes

If applicable, is the statistical analysis and its interpretation appropriate?

Yes

Are all the source data underlying the results available to ensure full reproducibility?

Yes

Is the study design appropriate and is the work technically sound?

Yes

Are the conclusions drawn adequately supported by the results?

Yes

Are sufficient details of methods and analysis provided to allow replication by others?

Yes

Reviewer Expertise:

biostatistics, community engagement, environmental health, epidemiology, global health, health policy and management, and health promotion and socio-behavioural health sciences.

I confirm that I have read this submission and believe that I have an appropriate level of expertise to confirm that it is of an acceptable scientific standard.

# Reviewer report: Reviewer response for version 1

**DOI:** 10.5256/f1000research.167832.r319429

**Published:** 2024-09-10

**Authors:** Atrey J Pai khot

**Affiliations:** 1 Goa Dental College and Hospital, Bambolim, Goa, India; 2 KLE Academy of Higher Education and Research, Belagavi, India

The article is of good scientific interest. The selection of such an important research area demonstrates the author’s deep understanding of difficulties in health promotion among pregnant women. The findings presented in this article have the potential for decision-making in the context of oral health care in pregnant women. Well-conducted research with meticulous study design. Overall results are presented in a logical and comprehensible manner.

Is the work clearly and accurately presented and does it cite the current literature?

Yes

If applicable, is the statistical analysis and its interpretation appropriate?

Yes

Are all the source data underlying the results available to ensure full reproducibility?

Yes

Is the study design appropriate and is the work technically sound?

Yes

Are the conclusions drawn adequately supported by the results?

Yes

Are sufficient details of methods and analysis provided to allow replication by others?

Yes

Reviewer Expertise:

Infectious Disease Control and Prevention / Public Health / Dental Public Health /  Epidemiology / Alternative Medicine / Dentistry / Randomized Clinical Trials / Meta-Analysis / Tobacco Control / Community Health Care / Special Health Care/ Health Education and Promotion

I confirm that I have read this submission and believe that I have an appropriate level of expertise to confirm that it is of an acceptable scientific standard.

# Reviewer report: Reviewer response for version 1

**DOI:** 10.5256/f1000research.167832.r319433

**Published:** 2024-09-05

**Authors:** Amit Vasant Mahuli

**Affiliations:** 1 Department of Public Health Dentistry, Rajendra Institute of Medical Sciences, Ranchi, Jharkhand, India

Summary 

 Its a well reported study. However, the following observations can be considered: 

 1. Some text is redundant from introductory lines of methodology and inclusion criteria's 

 2. Even though the sample size is calculated, the sample size is small. The results are conclusive in favour of the motivational interviewing, but there are inherent advantages and disadvantages to both methods. Recommendations and limitations of the study are very well mentioned.

 3. The loss to follow up analysis, either Intention treat or Per-protocol analysis used, has to be justified 

 4. The data on accessibility to health care service and location, either urban or rural, can give better insights in relation to the title.

 Overall, it is a well written article highlighting the importance of oral health awareness and prevention of oral health disease in pregnant women

Is the work clearly and accurately presented and does it cite the current literature?

Yes

If applicable, is the statistical analysis and its interpretation appropriate?

Yes

Are all the source data underlying the results available to ensure full reproducibility?

Yes

Is the study design appropriate and is the work technically sound?

Yes

Are the conclusions drawn adequately supported by the results?

Yes

Are sufficient details of methods and analysis provided to allow replication by others?

Yes

Reviewer Expertise:

Public Health Dentistry, Oral Cancer, School Oral Health, Epidemiology of oral health and systematic review & meta-analysis

I confirm that I have read this submission and believe that I have an appropriate level of expertise to confirm that it is of an acceptable scientific standard.
